# A voting-based ensemble feature network for semiconductor wafer defect classification

**DOI:** 10.1038/s41598-022-20630-9

**Published:** 2022-09-28

**Authors:** Sampa Misra, Donggyu Kim, Jongbeom Kim, Woncheol Shin, Chulhong Kim

**Affiliations:** 1 Department of Convergence IT Engineering, Pohang University of Science and Technology, Pohang, 37673 South Korea; 2 NAND Data Science Team, SK Hynix, Icheon, 17336 South Korea; 3 Department of Electrical Engineering, Mechanical Engineering, and also with the Medical Device Innovation Center, Pohang University of Science and Technology, Pohang, 37673 South Korea

**Keywords:** Computer science, Scientific data

## Abstract

Semiconductor wafer defects severely affect product development. In order to reduce the occurrence of defects, it is necessary to identify why they occur, and it can be inferred by analyzing the patterns of defects. Automatic defect classification (ADC) is used to analyze large amounts of samples. ADC can reduce human resource requirements for defect inspection and improve inspection quality. Although several ADC systems have been developed to identify and classify wafer surfaces, the conventional ML-based ADC methods use numerous image recognition features for defect classification and tend to be costly, inefficient, and time-consuming. Here, an ADC technique based on a deep ensemble feature framework (DEFF) is proposed that classifies different kinds of wafer surface damage automatically. DEFF has an ensemble feature network and the final decision network layer. The feature network learns features using multiple pre-trained convolutional neural network (CNN) models representing wafer defects and the ensemble features are computed by concatenating these features. The decision network layer decides the classification labels using the ensemble features. The classification performance is further enhanced by using a voting-based ensemble learning strategy in combination with the deep ensemble features. We show the efficacy of the proposed strategy using the real-world data from SK Hynix.

## Introduction

The semiconductor manufacturing process involves complex processes that form integrated circuits on the wafer surface. Manufactured wafers are first divided according to whether they have defects or not. Afterward, in order to analyze the wafers with defects, a binary wafer map is created where the defective chip on the wafer has a value of 1 and vice versa^1^. A specific pattern (e.g., cluster, scratch, edge, etc.) is usually formed based on those binary values on the wafer map^2^. However, no correlation has been established between the cause of the defect and the specific pattern. Therefore, semiconductor manufacturers are trying to find the cause of a defect by gathering wafer maps with similar patterns and identifying commonalities between defective wafers^3^.

Defect classification is the first step for collecting and analyzing wafer maps with similar patterns. In general, defect classification is done by humans which is time-consuming, laborious, and causes human error^4^. Nowadays, as the semiconductor manufacturing process has large amounts of samples, the importance of reducing the time with high accuracy in this classification process has also increased to improve inspection quality and reduce human resource requirements for defect inspection^5^.

Thus, automatic defect classification (ADC) of the wafer surface in less time and more accurately using deep learning (DL) is a welcome approach^6^. Here, we introduce a convolutional neural network (CNN)-based ensemble learning technique with voting for automatic defect classification, reducing analysis time and removing human error inconsistencies. Training CNN requires large datasets of labeled images with high computational costs, so we used transfer learning (TL) to solve the image shortage for training CNN models^7^. In addition, we adopted the ensemble learning method to further improve classification performance. Our proposed method was evaluated on real wafer map data from SK Hynix.

## Related work

### Conventional ADC methods

Semiconductor manufacturers have introduced ADC systems to reduce manufacturing and labor cost while improving product quality. In the past few years, the primary research areas now focus on wafer map feature extraction and defect pattern categorization using machine learning (ML) methods because of their robustness in the initial data deficiency events. The pre-defined hand-crafted features, such as edge features, surface texture, and pattern information, were first obtained manually from the wafer maps. Then ML techniques, such as support vector machine (SVM), random forest^8^, K-nearest neighbor (KNN), were used to classify wafer defects. Many techniques were employed for feature extraction e.g., geometry-based features^9^, representative features^10^, radon-based features^11^, texture features^12^, and density-based features^13^. A method for wafer map defect pattern recognition was proposed in^9^ by combining geometry-based and radon-based feature extraction, and then the SVM classifier was applied to classify the defect patterns. Yu and Lu^14^ presented a wafer map defect detection method using local and nonlocal linear discriminant analysis to discover intrinsic manifold information to characterize defect patterns. From these studies, it can be observed that current defect classification models based on ML need manually extracted features from the skilled semiconductor engineer. Therefore, the previously proposed ML-based models are generally expensive, inefficient, and time-consuming.

### Deep learning and ensemble learning methods

DL has recently shown great merits since it can automatically extract compact features from highly dimensional and complex data. The CNN model has demonstrated state-of-the-art performance classifying image data among different DL models. The CNN model has also been employed in the semiconductor industry: Nakazawa and Kulkarni^1^ employed a CNN model for wafer map classification. A CNN model was designed by^15^ to classify wafer map patterns for failure recurrence monitoring. Cheon et al.^6^ developed a CNN model to extract features for defect categorization. In order to classify defects in through-silicon through processes, a CNN-based model was developed in^7^. While the proposed DL methods have shown promising results, the fundamental disadvantage of these techniques is that they need more than a few thousand training data sets with precise ground-truth labelling. Thus, limited data set would lead to insufficient training of the DL network. The TL approach can alleviate this problem of inadequate training data in DL^16,17^. The network is first trained in the TL approach with an available large-scale dataset, e.g., ImageNet. The trained model is then fine-tuned using the limited dataset. A CNN based on the TL method is developed in^7,18^ for automatic defect classification. Yu et al.^19^ focused on the issue of not enough images with labels. They developed a semi-supervised DL-based TL method by utilizing features and labels in an adversarial network. However, these methods used a single deep network for semiconductor wafer defect classification, potentially limiting their ability to extract features learned by various CNN networks.

Ensemble learning has become one of the hot topics in ML as it overcomes the limitation of the individual model. Compared to the individual deep network, ensemble learning methods utilize a set of learning algorithms to obtain better classification results, improving the stability and robustness of the approach than the constituent learning algorithms alone. Saqlain et al.^20^ extracted geometry, density, and radon-based features from the raw wafer image and then trained four classification methods using extracted features. The ensemble soft voting technique then combined accuracy from these classifiers. Kang and Kang^21^ built a hybrid classifier by combining ML classifier and CNN for wafer map defect pattern classification. An integrated densely connected convolutional network (DenseNet) and the deep forest for wafer map defect-recognition model were developed in^22^. The performance of these ensemble learning methods is established on custom features, which are unsatisfactory for indicating the semiconductor wafer defect images.

In this study, we propose an ensemble method, where CNN models are pre-trained using the ImageNet dataset. Among several CNN models, ResNet18^23^, AlexNet^24^, and VGG16^25^ models are employed in this study. One superior classifier is created by combining three CNN models for excellent prediction performance. The CNN models are initially trained using a sizable dataset of naturally occurring image annotations (ImageNet)^24^. Then, these models are fine-tuned using annotated semiconductor wafer defect dataset. The models are ensembled in two ways: first by combining features and then based on voting. The following sections give specifics about our implementation and test findings.

## Methods

### Deep ensemble feature framework

**Figure 1:**
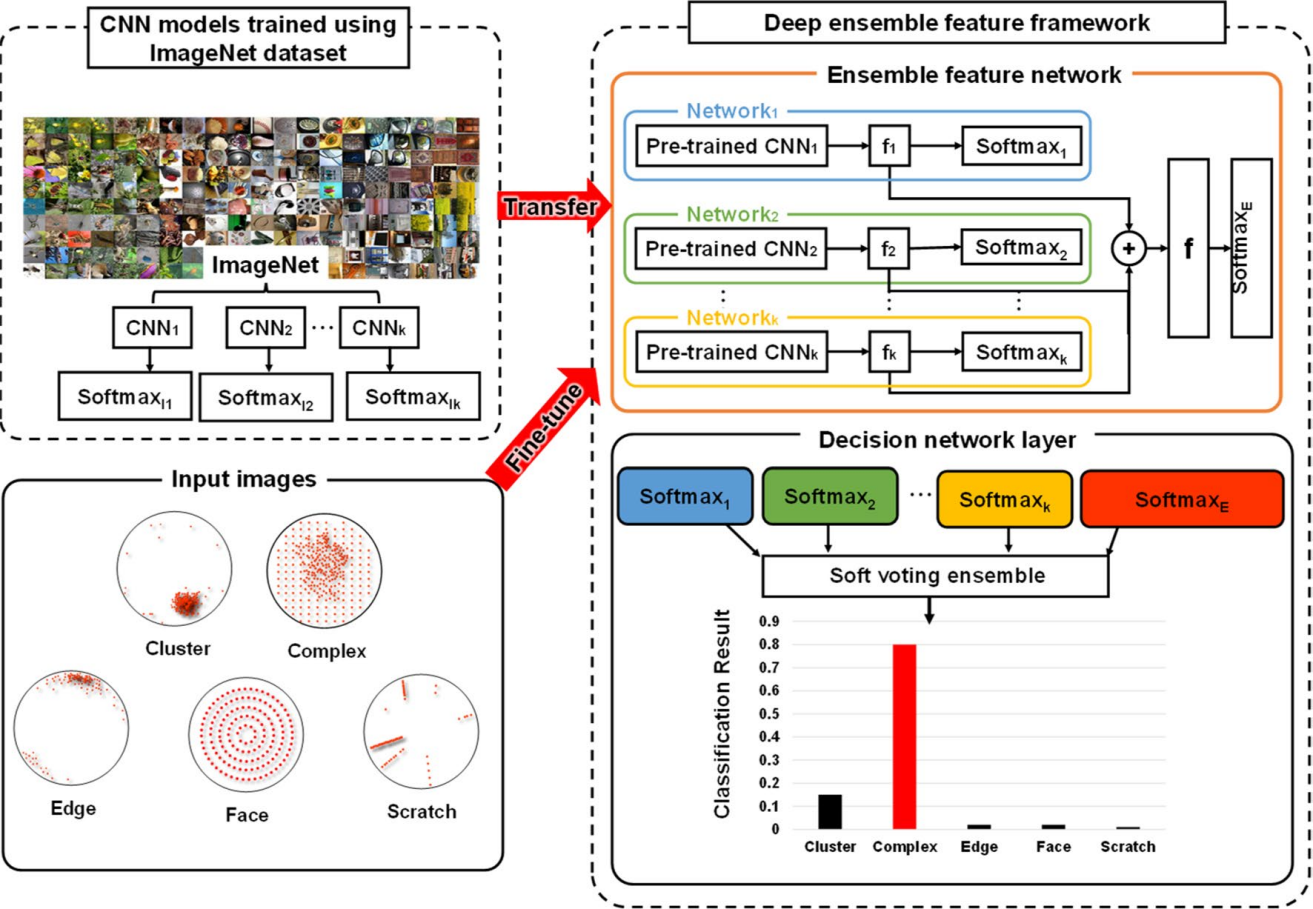
Schematic of deep ensemble feature framework. The framework is comprised of ensemble feature network and the decision network (softmax layer). The ensemble feature network includes *k* pre-trained CNN models using ImageNet data, and each CNN model provides the feature for classification. Ensemble feature is computed by concatenating features from different CNN models which acts as one of the inputs of the decision layer for the semiconductor wafer defect classification.

This section presents the proposed deep ensemble feature framework (DEFF) for wafer defect detection. The whole ensemble framework is shown in Fig. 1. Let $X=\left\{\left({x}_{i},{c}_{i}\right)1 \le i \le N\right\}$ be the dataset comprising of *N* training images with the corresponding class label *c*_*i*_ = *{1, 2, …, C},* where *C* is the total number of classes. The DEFF contains *K* different CNN models with fully connected (FC) layers and softmax layers. The proposed ensembled model can ensemble the random number of CNN models. However, we could only load three CNN (*k* = 3) models instantaneously due to the physical memory limits of the GPU card. The output of the last FC layer of the *k*^*t*^*h* CNN produces the deep features *f*_*k*_ for *kth* CNN model. A deep ensemble feature *f* is defined as *f* = *[f*_*1*_*, f*_*2*_*, … f*_*k*_*]*, which consists of all the deep features. In each epoch, the forward propagation is accomplished to produce features from each CNN model and the ensemble feature *f* is computed by concatenating these features. The decision network layer predicts the label of test images based on the voting ensemble features using *y*_*n*_ = *O(f*_*1*_*, f*_*2*_*, … f*_*k*_*, f)*.

### Deep CNN ensemble based on voting

A voting ensemble method can be divided into majority or hard voting and soft voting. The hard voting ensemble (HVE) can also be different types based on how the ensemble model selects class *c*: when all classifiers predict class as *c*; half of the base classifiers (majority) predict class as *c*. However, it is not optimal when the odd number of base classifiers exist and also when outputs of classifiers are independent. In our work, we use a soft voting ensemble (SVE), where the probability value is used instead of class labels for the ensemble. The output class is predicted by the average of the probability values. This approach provides more flexibility and fine-grained results than majority voting.

### Description of employed CNN models

In this study, we employed three state-of-the-art CNN models: deep residual network-18 (ResNet18)^23^, AlexNet^24^, and VGG16^25^ models. These popular CNN models have been widely used in many applications and show their robustness. Many researchers^26–28^ showed they have achieved the highest classification accuracy using these models for wafer map defect pattern identification. The network architecture of these models is shown in Supplementary Fig. 1. The ResNet18 model proposed by He et al.^23^, has one 7 × 7 convolutional layer, 5 residual blocks, and one fully connected (FC) layer. There are two regular residual blocks (Res block1) and three residual blocks with 1 × 1 convolution (Res block2). Each residual block contains two 3 × 3 convolutional layers, two batch normalization layers, and one ReLU layer. The AlexNet model, developed by Alex Kriszhevsky^24^ comprises five convolutional layers and the FC layers. After the first, second, and fifth convolutional layers, max-pooling layers are applied to reduce overfitting. The fifth convolutional layer (after max and avg pooling) is connected to the FC layers. The VGG16 model, developed by the Oxford Visual Geometry Group^25^, consists of 13 convolutional layers, five pooling layers, and three FC layers. For higher accuracy, a ReLu activation was employed for each convolutional layer.

All three models were pre-trained using the ImageNet dataset. The decision layers (softmax_I1_, softmax_I2_, … , softmax_Ik_) of these models were removed since these pre-trained CNN models aimed to classify 1000 classes. The features produced by these models were then concatenated to the ensemble feature, which served as the input of the softmax_E_ layer in ensemble feature network. The classification outcomes based on the deep ensemble features are computed as one of the inputs of the decision network layer. The final classification result is computed using a voting-based ensemble learning strategy from softmax_1_, softmax_2_, … , softmax_k_, softmax_E_. The convolution part was used for feature extraction. The outputs from the FC layer are directly utilized as feature descriptors for classification. The outputs of each layer are called features, and features from various layers have distinct significance. Local image features are extracted from the lower layer, and more semantic features are extracted from higher layers by convolution.

## Experiments

### Dataset

**Figure 2:**
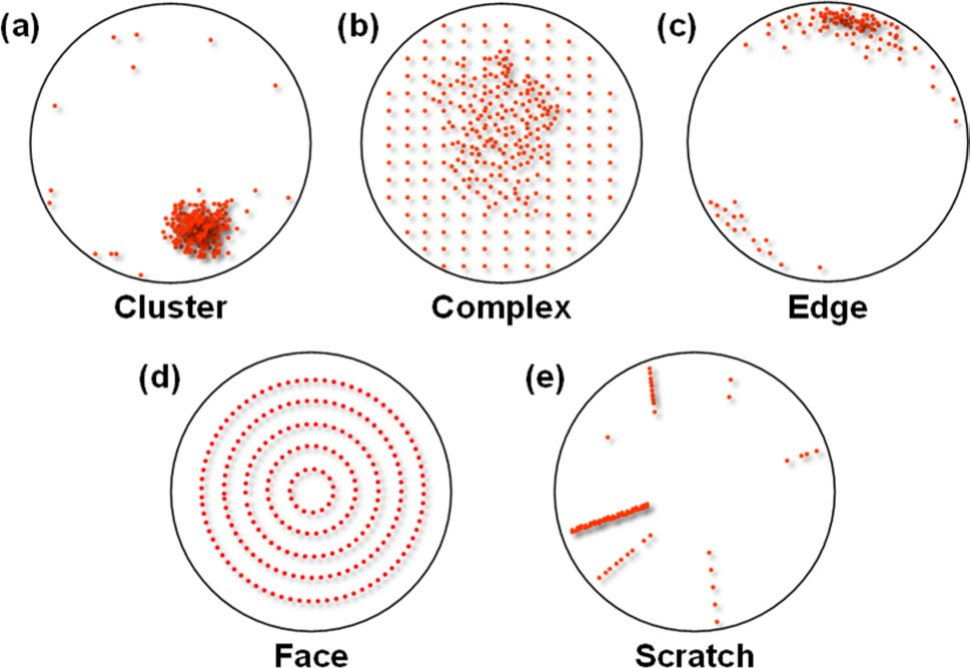
Typical examples of 5 wafer surface defect classes: (**a**) Cluster, (**b**) Complex, (**c**) Edge, (**d**) Face, and (**e**) Scratch.

The dataset employed in this study was obtained from the semiconductor manufacturing process of SK Hynix. An experienced engineer determined the wafer maps' class labels. There were a total of 2690 images and were divided into 5 classes, as shown in Table 1 (cluster: 500, complex: 141, edge: 395, face: 519, scratch: 1135). The sample defect images for each class are shown in Fig. 2.

**Table 1:** Class distribution of the dataset (Train, Validation, and Test sets).

Class name	Availablesamples	Training	Validation	Test
Cluster	500	320	80	100
Complex	141	90	23	28
Edge	395	253	63	79
Face	519	332	83	104
Scratch	1135	726	182	227
Total	2690	1722	430	538

### Experimental setting

The dataset was split randomly as follows: 80% for the training + validation and 20% for the testing for each class. The train + validation set was again randomly split as 80% for the training and 20% for the validation using fivefold cross-validation. This fivefold cross-validation process was repeated 4 times to generate 5 × 4 = 20 train and validation sets to statistically validate the model. The model could be evaluated using the validation set. However, it could result in overfitting. So, the test set, which is completely unknown during training time, is used to evaluate the model for avoiding the overfitting problem and showing the model robustness. The splitting process is shown in Supplementary Fig. 3. The number of images for train, validation, and test sets for all five defect classes are shown in Table 1. The total number of images in the train set, validation set, and test set was 1722, 430 and 538, respectively.

We implemented the DL models using PyTorch as the back-end programming language on a server that contained a total of 8 Dell PowerEdge MX740c blade servers. There were 500 training epochs in total. An early stopping criterion was also implemented, i.e., stop training and have the weights from the best epoch recovered from memory if the validation loss does not reduce across 50 successive epochs. We used the most widely used saqldens-entropy loss function and the stochastic gradient descent (SGD) optimizer^29^ for training. The batch size, learning, and momentum were 32, 0.0001, and 0.8, respectively. The size of all the training and testing images been changed to 224 × 224.

In order to increase the training dataset, we also used a standard augmentation technique. As an augmentation technique, we used random cropping, 1° rotations, and horizontal flipping here.

### Evaluation metrics

In the multi-label classification problem, various metrics listed in Supplementary Table I can be used as evaluation indicators. The most commonly used metric is accuracy. However, accuracy is generally effective when the data is balanced^30^. The F1-score can measure performance even in the imbalanced data. Since the F1-score is based on the harmonic average and not on a simple average, it gives a penalty for a large value. With this principle, even if there is an imbalanced class with a large dataset, such as scratch, it is possible to effectively measure the performance of the model. In most real-life classification problems, imbalanced class distribution is prevalent, so the F1-score should be considered in evaluating the model. Macro is the average value without considering label imbalance by giving the same weight to all classes. The weighted average considers the amount of data in each class.

## Results

### Pre-trained models versus without pre-trained models

To overcome the limited data set issue, we used TL approach. We first trained six networks: ResNet18^23^, AlexNet^24^, and VGG16^25^, DenseNet121^31^, GoogLeNet^32^, and SqueezeNet^33^ with the training set. These models are well known and have performed well when adapted to the classification of defected patterns in wafer bin maps^26–28,34,35^. The key salient features of these models are shown in Supplementary file. We compared the performance of six networks with TL and without TL. Models with TL have pre-trained weights from large datasets, and we used them to train our new models. On the other hand, the models without TL have randomly initialized weights without pre-trained weights. In other words, models without TL don't use ConvNets pre-trained with large datasets of ImageNet.

**Figure 3:**
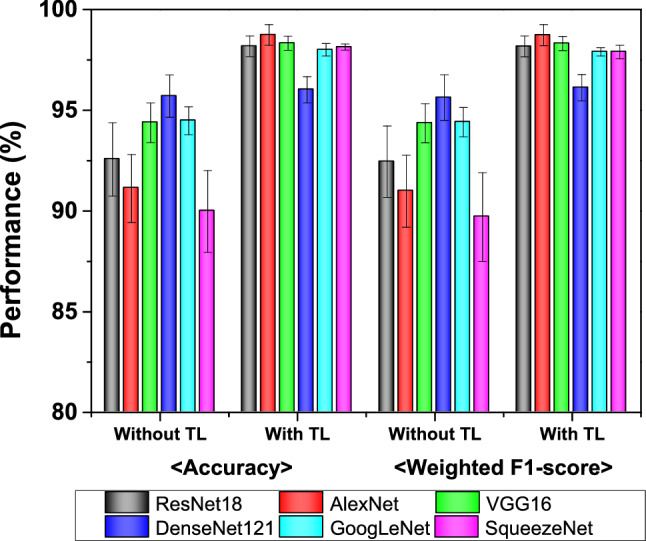
The graph representation of classification performance (mean) for each model without TL and that with TL. Error bars represent standard deviation (SD).

The classification performance in terms of accuracy and weighted F1-score on the test dataset with and without using TL is shown in Fig. 3. As shown in Fig. 3, we can see that the overall performance increased when TL was used compared to without TL. The accuracy increased from 92.56 to 98.42%, 91.12 to 98.31%, 94.38 to 98.11%, 95.7 to 96.02%, 94.48 to 98.0% and 89.98 to 98.14%, for ResNet18, AlexNet, VGG16, DenseNet121, GoogleNet, and SqueezeNet models, respectively. The results confirm that even when the data is limited, the TL method can improve performance.

### Various pre-trained CNN models

**Figure 4:**
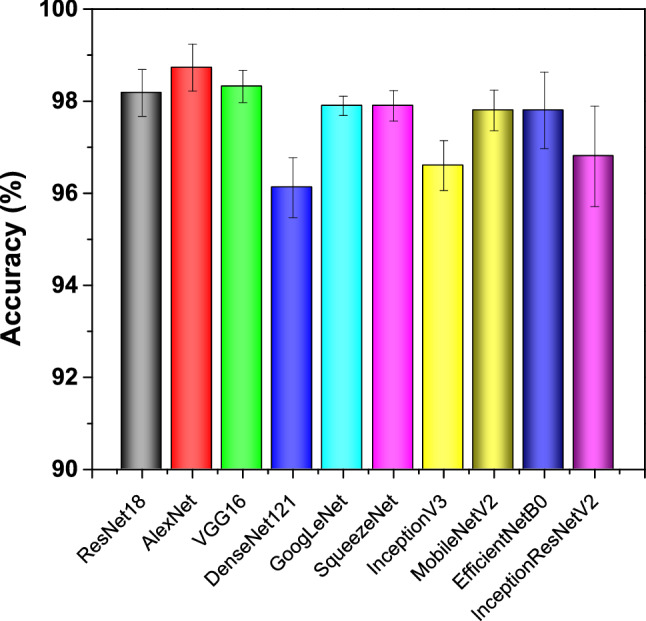
The graph representation of classification accuracy (mean) for pre-trained CNN models. Error bars represent standard deviation (SD).

The classification performance in terms of accuracy for various pre-trained (ImageNet) CNN models is shown in Fig. 4. The classification accuracy of 10 CNN models, namely, ResNet18^23^, AlexNet^24^, and VGG16^25^, DenseNet121^31^, GoogLeNet^32^, SqueezeNet^33^, InceptionV3^36^, MobileNetV2^37^, EfficieneNetB0^38^, InceptionResNetV2^39^ are compared. For some CNN models, the performance is not that much significant when adapted to another field, although classification accuracy of the CNN models is higher using ImageNet dataset. For example, DenseNet (2016) model is a more recent and advanced model than AlexNet (2012), ResNet (2015), and VGG (2014) models. However, the performance of the DenseNet model is inferior to the other models using our dataset.

### Ensemble versus single models

We applied ensemble learning to further improve the classification performance of pre-trained CNN models. The ensemble of the different models complements each other and overcomes the limitations of the single model. The ensemble architectures can be incredibly useful in acquiring different features. The proposed ensembled model can ensemble the random number of CNN models and modify the parameters of these models in an end-to-end trainable manner. However, we could only load three CNN models instantaneously due to the physical memory limits of the GPU card. The 3 best CNN models shown in Fig. 3 are ResNet18 (R), AlexNet (A), and VGG16 (V) were used as sub-models for ensemble learning.

The classification performance of pre-trained single models and ensemble models is shown in Table 2. The union of each CNN's acronyms represents the combination of CNNs. For example, R + A + V implies ResNet18, AlexNet, and VGG16 were used in our proposed method for the end-to-end training. The classification performance of the ensemble model based on the proposed DEFF method as well as HVE and SVE are superior to that of single models. It is important to note that the performance of the proposed DEFF model using 3 models is superior to the DEFF model using 2 models. Finally, after applying ensemble voting with the proposed DEFF, we obtained an accuracy of 99.15%. The accuracy of 99.15% implies except for 4 images all were classified accurately. The confusion matrix is shown in Supplementary Fig. 2.

**Table 2:**
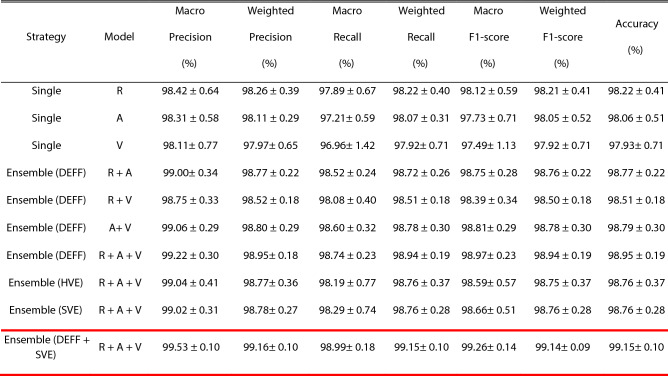
Classification performance (mean and SD) using different strategies from 20 independent runs.

Three CNNs including ResNet18 (R), AlexNet (A), and VGG16 (V) are applied. The Highest Performance Values are Highlighted in the red box in the table.

### Ablation studies

**Figure 5:**
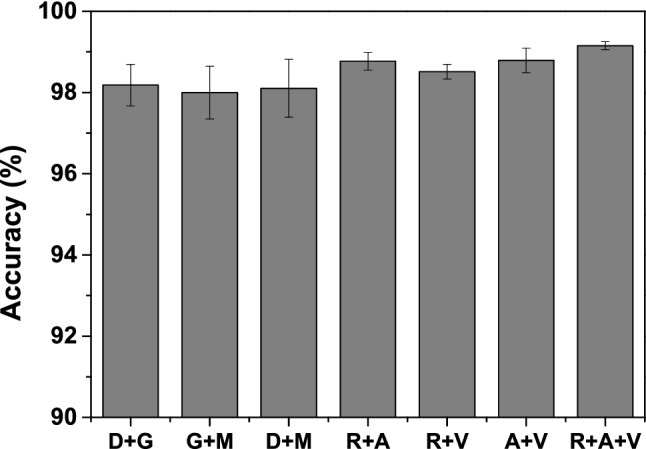
The graph representation of classification accuracy (mean) with different combinations of CNN models.

Here, we performed various combinations of CNN models for ablation studies. The accuracy of the ablation studies with different combinations of CNN models (e.g., DenseNet121 (D), GoogLeNet (G), MobileNetV2 (M)) is shown in Fig. 5. From Fig. 5, we can see that when we combine two CNN models, the best and the second-best accuracy values are obtained for A + V and R + A models, respectively. Nevertheless, the ensemble method with three CNNs (R + A + V) still accomplished the best accuracy value. It is worth noting that if we increase the number of CNNs, classification accuracy may be improved more.

### Robustness

The robustness of the proposed method is validated by its superior performance on publicly available datasets. German TILDA defect database^27,40,41^ (https://www.aitex.es/afid/ TILDA-C1R1^41^, TILDA-C2R2^41^, TILDA-C2R3^41^), MT^42^ (https://github.com/abin24/Magnetic-tile-defect-datasets), and AITEX^43^ (https://www.aitex.es/afid/) datasets are employed to evaluate the model. The classification performance in terms of accuracy of these datasets is shown in Table 3. The proposed ensemble model shows the overall values of accuracy 97.505, 90.00, 92.50, 94.90 and 98.90% using TILDA-C1R1, TILDA-C2R2, TILDA-C2R3, MT, and AITEX datasets, respectively. It is worth noting that the ensemble model outperforms other single CNN models. Among 3 CNN models, the performance of VGG16 is superior to ResNet18 and AlexNet models.

**Table 3:** Classification performance using different datasets.

Dataset/Model	Accuracy (%)				
	TILDA-C1R1	TILDA-C2R2	TILDA-C2R3	MT	AITEX
ResNet18	95.00	80.00	87.50	89.54	91.50
AlexNet	75.00	72.50	80.00	84.64	92.00
VGG16	90.00	82.50	87.50	91.40	95.50
Proposed (Ensemble)	97.50	90.00	92.50	94.90	98.90

### Statistical analysis

All the results were statistically validated for all the test cases using the two-tailed paired *t*-test^44^ considering the null hypothesis that the performance of the two models was equivalent. The statistical analysis is shown in Table 4. The *p* values are provided for a 95% confidence interval, and the significance is denoted by two signs: * indicates that the model performed significantly better (i.e., *p* 0.05, rejecting the null hypothesis), and indicates that the performance of two models was equivalent (i.e., *p* > 0.05 could not be used to reject the null hypothesis).

**Table 4:** Statistical analysis of ensemble models and single models from 20 runs.

	R + A	R + V	A + V	R + A + V	HVE	SVE	DEFF + SVE
R	*	*			*	*	*
A	*		*		*	*	*
V		*	*		*	*	*
R + A		≈	≈	*	*	*	*
R + V	≈		≈	*	*	*	*
A + V	≈	≈		*	*	*	*
R + A + V					*	*	*
HVE						≈	*
SVE					≈		*

Here, * denoting the model’s performance is noticeably better than that of other models and ≈ indicating the performance of two models is equivalent.

### Saliency map

**Figure 6:**
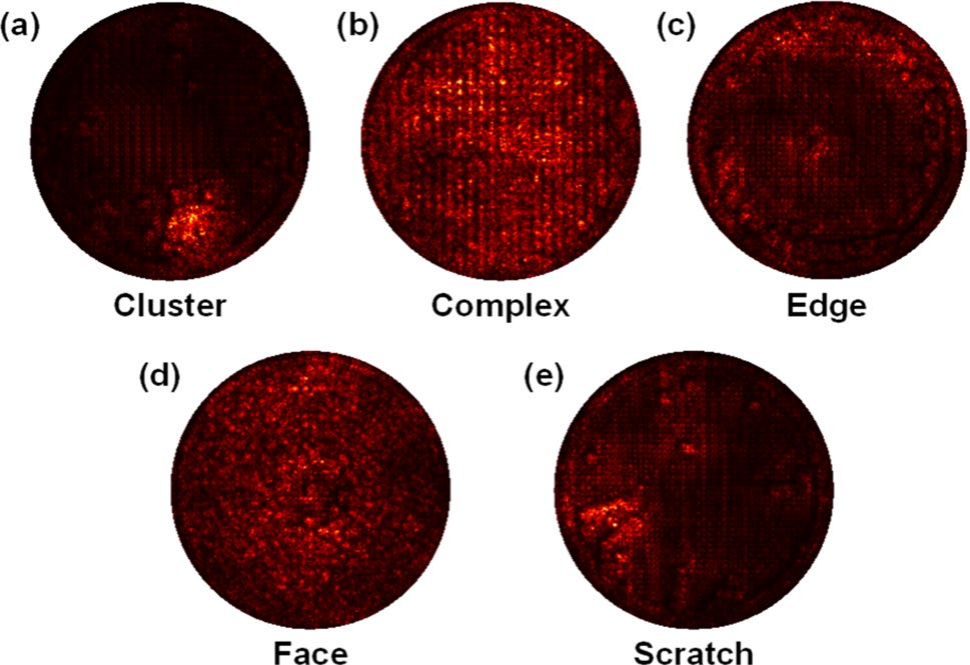
Saliency maps of 5 defect classes: (**a**) Cluster, (**b**) Complex, (**c**) Edge, (**d**) Face, and (**e**) Scratch.

The saliency maps of five various defect image classes are demonstrated in Fig. 6. It illustrates which part of the images is used by the CNN model for classifying defects. It is obtained by computing the gradient values of the output class score to input image pixel intensity. For example, a test image of a given class is input to a trained CNN model, the associated output class is predicted from the classification layer. Then, the gradient of the predicted class for each input pixel is obtained by performing backpropagation. The map shows the gradient values of all input pixels. The more a pixel is activated for categorization, the higher its gradient value. The detailed description of the saliency map is described in Simonyan et al.^45^. Figure 6 shows the region of the wafer where the defects were located. The trained CNN model focuses on the location of the bright pixel of the saliency map. This result shows that the proposed ensemble model successfully locates the positions of defect occurrences and captures high-quality classification features.

### Comparison with existing methods

Due to the difference in the quantity of test images and data sources, it is not possible to compare the performance of the proposed approach with other existing methods. In Table 5, we have summarized the performance in terms of accuracy, classifiers, and the architecture used in our proposed model, other existing traditional ML models^13,14^, DL models^1,2,6^, and ensemble-based^11,20,21,34^ classification models for wafer map pattern classification.

**Table 5:** Classification performance (accuracy) of proposed and existing methods.

Approach	Reference	Features/ Model	Classifier	Accuracy (%)
ML	Fan et al.^13^	Density, Geometry	SVM	88.22
	Yu and Lu^14^	Geometry, gray, texture, projection	JLNDA-FD	85.44
ML-Ensemble	Piao et al.^11^	radon transform, geometric	DT	78.48
	Saqlain et al.^20^	density, geometry, radon	SVE	95.86
CNN	Nakazawa and Kulkarni^1^	Wafer Map	CNN	98.20
	Saqlain et al.^2^	Wafer Map	CNN	96.20
	Cheon et al.^6^	Wafer Map	CNN	96.20
CNN-Ensemble	Kang and Kang^21^	Geometry, Density, Radon, Wafer Map	CNN, FNN	94.62
	Hsu and Chien^34^	Wafer Map	WMV	98.57
	**Proposed**	**Wafer Map**	**DEFF + SVE**	**99.15**

The best performance is shown in bold font. *ML* machine learning, *CNN* convolutional neural network, *SVM* support vector machine, *JLNDA* joint local and nonlocal linear discriminant analysis, *FD* fisher discriminative, *DT* decision tree, *SVE* soft voting ensemble, *FNN* feed-forward neural network, *WMV* weighted majority voting, *DEFF* deep ensemble feature framework.

### Time cost

Table 6 shows the time cost of single CNN models and proposed ensemble model. Although the computational effort is large for the implementation of CNN-based approaches, they are simple to use and can automatically capture useful features without specialized domain knowledge. Additionally, methodologies based on CNN are gaining popularity for classifying wafer defect patterns since they are highly accurate and outperform other ML-based techniques.

**Table 6:** Training time of the single and ensemble models.

Model	Training time (s)
VGG-16	1074.23
ResNet	307.41
AlexNet	119.37
Ensemble	925.64

## Discussion

The presented ensemble model offers excellent performance due to (1) fine-tuning learning features that are specific to our dataset, and (2) the ensemble of different models overcoming the limitations of the individual models.

Our results indicate that the shallower networks, e.g., AlexNet features are more generalizable and adaptable when transferred to a different domain. On the other hand, deeper networks, such as DenseNet features are more semantically optimized for natural images. In our study, DenseNet achieved higher accuracy than AlexNet for natural image classification, but the performance was lower for semiconductor wafer defect classification. As shown in Fig. 3, the improvement of classification accuracy for AlexNet with pre-trained data was 8% in comparison to without pre-trained AlexNet. In contrast, the accuracy of DenseNet improved only by 0.04% when pre-trained data was used.

The TL results tabulated in Table 2 showed the characteristics and strengths of the various CNN architectures. Three CNN models, i.e., including ResNet18, AlexNet, VGG16, achieved better classification performance than other CNN models for semiconductor wafer defect classification. Thus, in our ensemble approach, we employed these 3 models.

In order to achieve several features, ensemble architectures might be of great assistance. We are able to extract image features that are especially pertinent to the semiconductor defect images being classified thanks to the fine-tuning of the CNNs in our ensemble model. Herein, we observed that the ensemble model precisely classifies images that individual models often misclassify.

The proposed ensemble method gives a general architecture for ensembling any number of CNN models. Thus, the proposed method can learn more representative deep ensemble features to achieve better performance compared to the preliminary method.

**Figure 7:**
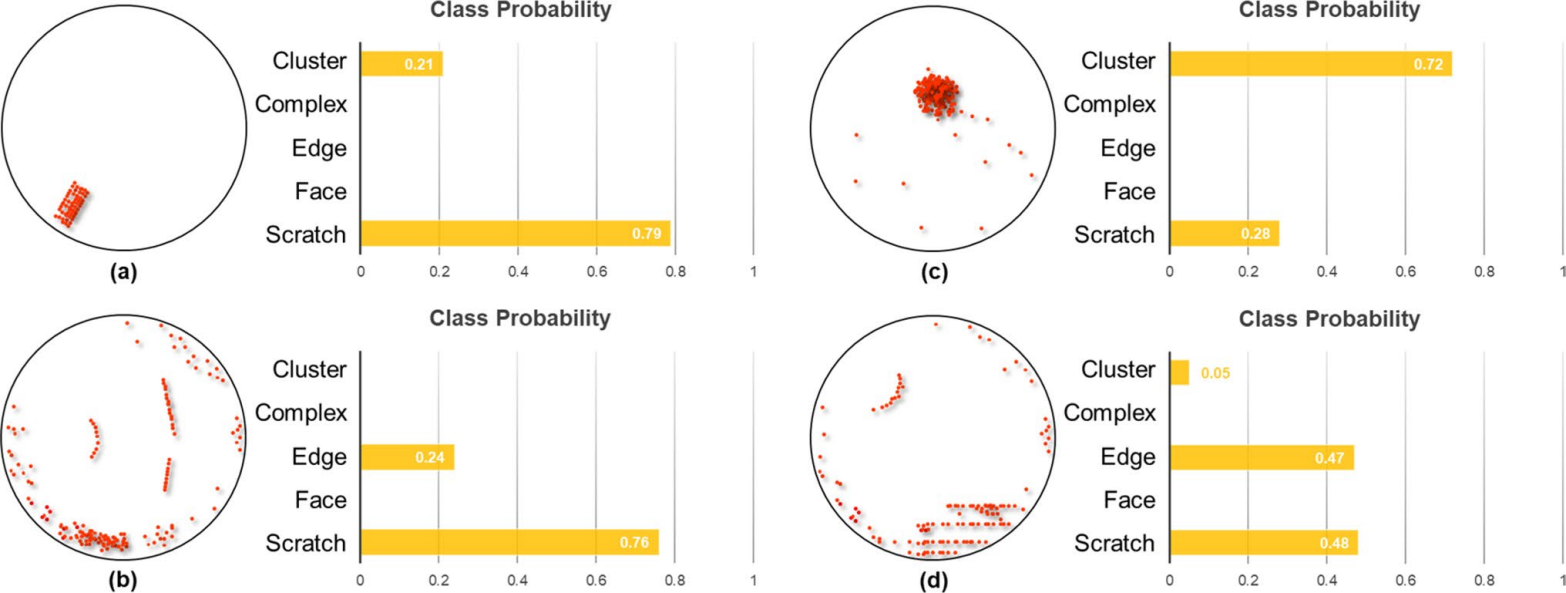
Examples of misclassified images: (**a**) actual class: Cluster, predicted class: Scratch, (**b**) actual class: Edge, predicted class: Scratch, (**c**) actual class: Scratch, predicted class: Cluster, (**d**) actual class: Edge, predicted class: Scratch*.*

Even if the proposed strategy is very effective, 4 images were misclassified. Details of the misclassification images and their corresponding predictive probability values (PPV) for each class are shown in Fig. 7. The average PPV values for the images that were correctly classified are ~ 0.98 for each class. However, for the misclassified images, the PPV values are ~ 0.72–0.79. The true class of the misclassified image is the cluster as shown in Fig. 7a, but it was misclassified as scratch. The class probability is 0.21 for cluster and 0.79 for scratch. The defect map of Fig. 7a is not formed by random particles gathered to form a typical cluster class image, instead line-based defects are gathered to form a cluster. Thus, the probability of scratch classification is high because of this defect map feature. In the case of Fig. 7b, the class is an edge, but it is misclassified as scratch. The probability for the actual class was 0.24, and the probability for the scratch was 0.76. The defect map of Fig. 7b contains defects distributed along the edge of the wafer, but the probability of scratch is high because the scratch-type defect is in the center part of the wafer surface. In the case of Fig. 7c, the true class is scratch, but it is misclassified as cluster. The probability for the scratch class was 0.28, and the probability for the cluster class was 0.72. The defect map of Fig. 7c is mainly due to incorrect labeling. Defect maps of Fig. 7a and b are due to combined defects of two classes. In the case of Fig. 7c, the main reason for scratch characteristics was not found. The wafer maps' class labels were determined by an experienced engineer, and human error is inevitable. The goal of our approach is also to overcome such human error through auto-classification. Figure 7d shows the misclassification example of edge, and the prediction is the scratch. The class probability for edge and scratch class was 47 and 48%, respectively. In the defect map of Fig. 7d, the defects are distributed along the edge, but the class probability for edge and scratch was 0.47 and 0.48, respectively, because the scratch type defect is in the center part of the image, similar to Fig. 7b.

Our method, while showing high performance, however, has few limitations. First, our ensemble approach has three CNN models, so it needs a very sophisticated computer for its implementation and requires a very high computational cost. Second, we used the same fine-tuning parameters for all CNN models and did not do any parameter optimizations. Third, we used the pre-trained data set from a different domain (i.e., ImageNet). Although the performance of cross-domain TL is excellent, it may not be the optimal choice.

We intend to enhance the suggested approach by incorporating class imbalance and data scarcity, which are real-world data concerns in semiconductor wafer defect analysis. We also intend to combine handcrafted and convolutional features to capitalize their respective strengths.

We plan to improve the performance of the ensemble classifier by giving the decision values of that classifier more weight. We are developing a weighted soft voting ensemble (WSVE) to improve the voting ensemble method. The weighted soft voting ensemble is defined as:

1 $$WSVE={w}_{1}*{CNN}_{1}\left(p\right)+ {w}_{2}*{CNN}_{2}\left(p\right)+ {\dots + w}_{K}*{CNN}_{K}(p)$$

where *w*_*k*_ and *CNN*_*k*_*(p)* are the weight and probability of the *kth* CNN model. The predicted class $\widehat{\mathrm{y}}$ of an image in the test set is computed by

2 $$\widehat{\mathrm{y}}=\mathrm{arg}\underset{j}{\mathrm{max}} \left[\sum_{i=1}^{m}\frac{{w}_{i}\times {p}_{i,j}}{m+1}\right]$$

where *m* is the number of models used for ensemble learning, *w* is the weight for each model, *p* is the probability, and function arg max returns the value of *j* such that the expression in parentheses in Eq. (1) is the maximum value. Here, we would use the weighted F1-score of each model as weight, and the model that achieves the best classification performance would be given double weight. Thus, when calculating the weighted average of *m* models in weighted voting, it is divided by *m* + 1 instead of *m*. We plan to evaluate this equation and then make various experimental attempts to find the optimal weights. We expect this approach would improve defect classification performance further.

## Conclusion

In this paper, we proposed a novel voting based DEFF for classifying wafer map defects. We built the classification model based on CNN and trained with an industrial real wafer map dataset. The vast majority of earlier evaluations of wafer defects utilized machine learning-based classification algorithms, which necessitated human feature extraction and many hyper parameter settings. On the contrary, the CNN model presented here has the ability to automatically extract useful features from different defect classifications. We have applied the data augmentation technique to enhance the number of images available to train the model. The proposed method simultaneously learns deep feature representations from CNN models, and the decision layer accomplishes better classification accuracy in an end-to-end trainable fashion. We also used soft voting after getting deep ensemble features to further improve the performance. In this implementation, we employed three CNN models including VGG16, AlexNet, and ResNet18 models. We showed the effectiveness of combining multiple CNN models for recognizing wafer map defect patterns through ablation studies. A more reliable automation of wafer map defect pattern classification is anticipated as a result of the increased classification performance.

## Supplementary Information

Supplementary Information.

## Data Availability

The data that support the findings of this study are available from SK Hynix, but restrictions apply to the availability of these data, which were used under license for the current study, and so are not publicly available. Data are however available from the authors upon reasonable request and with permission of SK Hynix.
