# Supplementary material for: A voting-based ensemble feature network for semiconductor wafer defect classification
Source: Sci Rep. 2022 Sep 28;12:16254. doi: 10.1038/s41598-022-20630-9 (PMC9519991; doi:10.1038/s41598-022-20630-9)
Supplement: Supplementary file 1 — Supplementary Information. [file 41598_2022_20630_MOESM1_ESM.docx]

Supplementary Information for:

**A Voting-based Ensemble Feature Network for Semiconductor Wafer Defect Classification**

Sampa Misra^1^, Donggyu Kim^1^, Jongbeom Kim^1^, Woncheol Shin^2^, and Chulhong Kim^1,3^, *Senior Member, IEEE*

***Corresponding author:** [chulhong@postech.edu](mailto:chulhong@postech.edu)

^1^Department of Convergence IT Engineering, Pohang University of Science and Technology, Pohang 37673, South Korea

^2^ NAND Data Science Team, SK Hynix, Icheon 17336, South Korea

^3^ Department of Electrical Engineering, Convergence IT Engineering, Mechanical Engineering, and also with the Medical Device Innovation Center, Pohang University of Science and Technology, Pohang 37673, South Korea

**Table of contents**

**Supplementary Figures**

Supplementary Fig. 1. The network structures of ResNet18, AlexNet, and VGG16 models2

Supplementary Fig. 2. Confusion matrix of the proposed model3

Supplementary Fig. 3. Splitting process of the dataset.3

**Supplementary Tables**

Supplementary Table 1. Evaluation metrics for multi-label classification4

**Supplementary** **Appendix**

Supplementary Appendix 1. CNN models and their main salient features5

**Supplementary Fig. 1. The network structures of ResNet18, AlexNet, and VGG16 models.** Conv stands for convolutional layer, ReLu stands for rectified linear unit activation. The fully connected (FC) layer has five outputs (Cluster, Complex, Edge, Face, and Scratch).


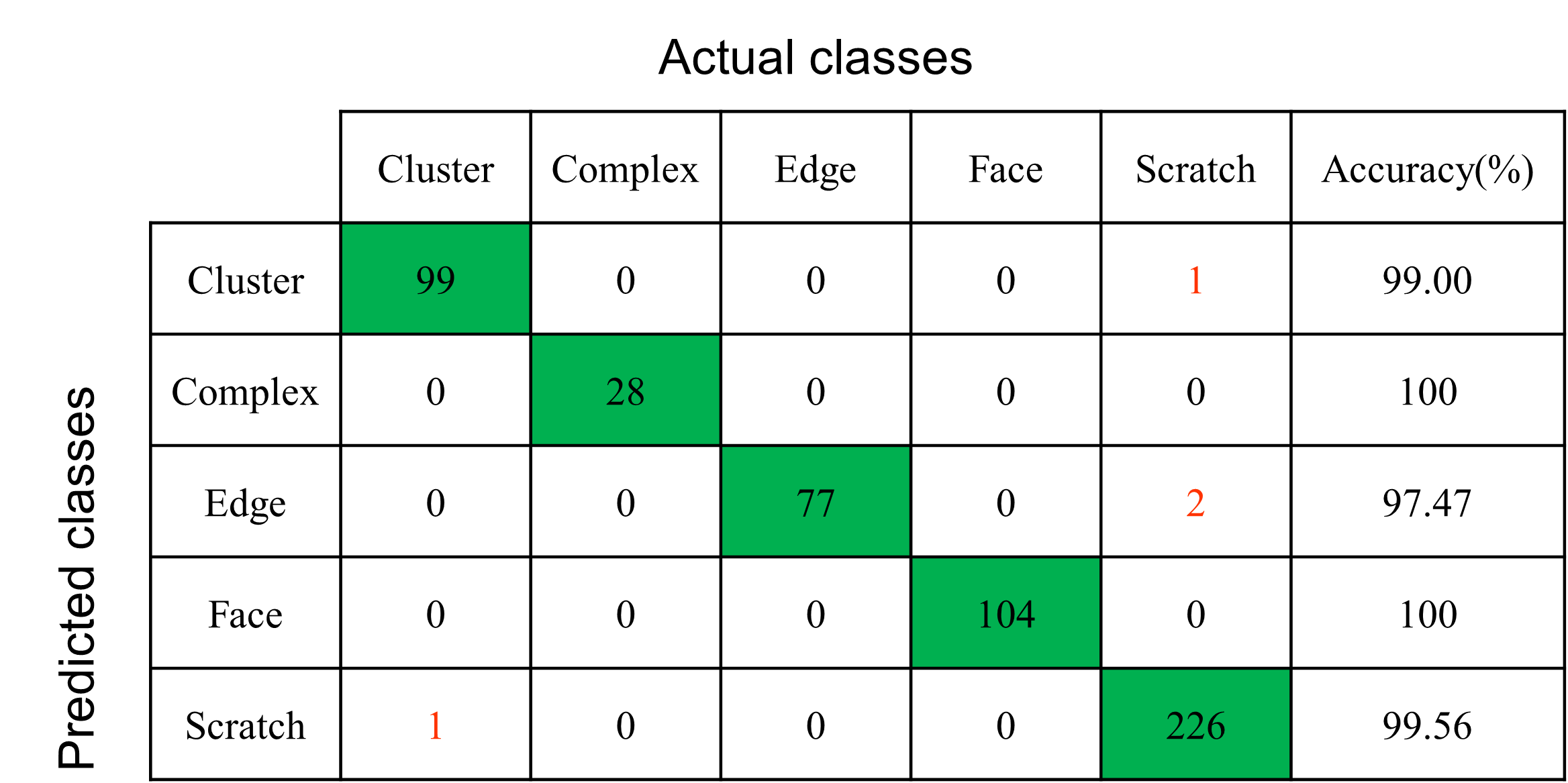


**Supplementary Fig. 2.** Confusion matrix of the proposed model.

**Supplementary Fig. 3.** Splitting process of the dataset.

**Supplementary Table I.** Evaluation metrics for multi-label classification. *TP*: true positive, *FP*: false positive, *FN*: false negative, *TN*: true negative, *K*: the number of classes, *n*: the number of images in each class, *N*: the number of total test images.

| **Metrics** | **Formula** | **Evaluation Focus** |  |
| --- | --- | --- | --- |
| Precision | $\mathrm{precision}_{j}=\frac{{TP}_{j}}{{TP}_{j}+{FP}_{j}}$ | How accurate the detected results are that is, how many real objects are included in the detection results. |  |
| Recall | $\mathrm{recall}_{j}=\frac{{TP}_{j}}{{TP}_{j}+{FN}_{j}}$ | How well it classifies objects without missing them |  |
| F1-score | ${(F_{1}-score)}_{j}=2 \times\frac{{precision}_{j}\times{recall}_{j}}{{precision}_{j}+{recall}_{j}}$ | Harmonic mean between precision and recall values |  |
| Macro precision | $\sum_{j=1}^{K} \frac{{precision}_{j}}{K}$ | The average of per-class precision |  |
| Macro recall | $\sum_{j=1}^{K} \frac{{recall}_{j}}{K}$ | The average of per-class recall |  |
| Macro F1-score | $\sum_{j=1}^{K} \frac{{(F_{1}-score)}_{j}}{K}$ | The average of per-class $F_{1}$-score |  |
| Weighted precision | $\sum_{j=1}^{K} \frac{n_{j}\times{precision}_{j}}{N}$ | The weighted average of per-class precision |  |
| Weighted recall | $\sum_{j=1}^{K} \frac{n_{j}\times\mathrm{recall}_{j}}{N}$ | The weighted average of per-class recall |  |
| Weighted F1-score | $\sum_{j=1}^{K} \frac{n_{j}\times{(F_{1}-score)}_{j}}{N}$ | The weighted average of per-class $F_{1}$-score |  |
| Accuracy | $\sum_{j=1}^{K} \frac{{TP}_{j}}{N}$ | Overall effectiveness of a classifier | |

**Supplementary Appendix 1. CNN models and their main salient features**

The architecture of ResNet18, AlexNet, VGG16, DenseNet121, GoogLeNet, and SqueezeNet are well established and have shown good performance when adapted to the classification of defected patterns in wafer bin maps [1-5] The main salient features of these models are:

**ResNet (Shortcut connections):** All neurons do not have to activate in every epoch for the ResNet model. It focuses on learning novel features rather than trying to learn a previously learned feature again. As a result, training takes much less time and accuracy is increased.

**AlexNet (Deeper):** Since AlexNet has an eight-layer deeper design, it can extract features more effectively. This network used ReLu activation function which does not limit the output, unlike other activation functions.

**VGG (Fixed-size kernels):** It makes significant improvements by sequentially replacing the large kernel-sized filters with many 3×3 kernel-sized filters. It benefits from excellent adaptability and high precision.

**DenseNet (Multi-layer feature concatenation):** It offers a variety of compelling benefits, including the elimination of the vanishing-gradient issue, improved feature propagation, promoted feature reuse, and significantly decreased parameter requirements.

**GoogLeNet (inception module):** It tries to improve the computational efficiency by using an inception module as the basic layer and stacking other layers of one another to apply parallel filtering on input from the layer before.

**SqueezeNet (Fire Module):** It reduces the number of network weights by using a 1×1 filter instead of a 3×3 filter, decreasing the number of input channels to 3×3 filters, and replacing the fully connected dense layers with a convolution layer.

[1] H. Kahng and S. B. Kim, "Self-supervised representation learning for wafer bin map defect pattern classification," *IEEE Transactions on Semiconductor Manufacturing,* vol. 34, no. 1, pp. 74-86, 2020.

[2] W. Shin, H. Kahng, and S. B. Kim, "Mixup-based classification of mixed-type defect patterns in wafer bin maps," *Computers & Industrial Engineering,* vol. 167, p. 107996, 2022.

[3] J. A. Mat Jizat, A. P. Abdul Majeed, Z. Taha, E. Yuen, and S. X. Lim, "Evaluation of the Transfer Learning Models in Wafer Defects Classification," in *Recent Trends in Mechatronics Towards Industry 4.0*: Springer, 2022, pp. 873-881.

[4] C.-Y. Hsu and J.-C. Chien, "Ensemble convolutional neural networks with weighted majority for wafer bin map pattern classification," *Journal of Intelligent Manufacturing,* pp. 1-14, 2020.

[5] P. Bhatnagar, T. Arora, and R. Chaujar, "Semiconductor Wafer Map Defect Classification Using Transfer Learning," in *2022 IEEE Delhi Section Conference (DELCON)*, 2022, pp. 1-4: IEEE.
